# L2pB1 Cells Contribute to Tumor Growth Inhibition

**DOI:** 10.3389/fimmu.2021.722451

**Published:** 2021-09-23

**Authors:** Varuna Shibad, Ali Bootwala, Changchuin Mao, Hanna Bader, Hung Vo, Esther Landesman-Bollag, Conrad Guo, Angel Rubio, Richard Near, Wenda Gao, Sreekar Challa, Vennela Chukka, Jeffrey Gao, Avery Kelly, Tamar Landesman, Tyler VanHelene, Xuemei Zhong

**Affiliations:** ^1^ Hematology Oncology Section, Department of Medicine, Boston University School of Medicine and Boston Medical Center, Boston, MA, United States; ^2^ Department of Graduate Medical Studies, Boston University School of Medicine, Boston, MA, United States; ^3^ Antagen Institute for Biomedical Research, Boston, MA, United States; ^4^ Department of Pharmacology, Boston University School of Medicine, Boston, MA, United States; ^5^ Sharon High School, Sharon, MA, United States; ^6^ Lynbrook High School, San Jose, CA, United States; ^7^ Brookline High School, Brookline, MA, United States

**Keywords:** L2pB1 cells, natural IgM, 3D tumor spheroids, melanoma, colon cancer, lipoptosis

## Abstract

Natural IgM (nIgM) antibodies play critical roles in cancer immunosurveillance. However, the role of B-1 B cells, the lymphocytes that produce nIgM, remains to be elucidated. L2pB1 cells, a subpopulation of B-1 B cells, have a unique poly-self-reactive nIgM repertoire and are capable of phagocytosis, potent antigen presentation, and immunomodulation. Using an inducible knock-in and knockout mouse model, we investigated the effect of the loss of L2pB1 cells in a B16F10 melanoma model. Our results show active tumor infiltration of L2pB1 cells in wild type mice, and conversely, depletion of L2pB1 cells results in larger tumor mass and increased angiogenesis. *In vitro* analysis revealed that L2pB1 cells contribute to the growth inhibition of melanoma cells in both 2D cell culture and 3D tumor spheroids. Similar effects were observed in an MC38 murine colon cancer model. Moreover, our data suggest that one of the ways that L2pB1 cells can induce tumor cell death is *via* lipoptosis. Lastly, we tested whether L2pB1 cell-derived monoclonal nIgM antibodies can specifically recognize tumor spheroids. Nine of the 28 nIgM-secreting L2pB1 clones demonstrated specific binding to tumor spheroids but did not bind control murine embryonic fibroblasts. Our study provides evidence that L2pB1 cells contribute to cancer immunity through their unique nIgM repertoire, tumor recognition, and lipoptosis. Taken together, because of their ability to recognize common features of tumors that are independent of genetic mutations, L2pB1 cells and their nIgM could be potential candidates for cancer treatment that can overcome tumor heterogeneity-associated drug resistance.

## Introduction

The existence of immune surveillance of cancer is evidenced by the increased spontaneous malignancies in both humans and animals with immune deficiency or immune suppression (1, 2). Natural IgM in healthy human sera has been reported to be cytotoxic to neuroblastoma cells (3). Recent studies show that higher numbers of tumor-infiltrating-B lymphocytes (TIL-B) are associated with better prognosis (4, 5) and TIL-B cells were found to recognize glycolipids exposed during tumor cell apoptosis (6, 7). These results indicate that natural antibody-producing B cells might play a key role in the immune surveillance of cancer that not only targets new, emerging cancers, but also may be actively involved in controlling established ones.

It is reported that the majority of natural antibodies in mice are produced by a special subgroup of CD5^+^ B lymphocytes, termed B-1a B cells (8). These IgM antibodies recognize “danger-associated” molecular patterns (DAMPs) and molecular patterns displayed on the surface of pathogens or stressed cells (9). During apoptosis, for instance, phosphorylcholine (PC), the headgroup of phosphatidylcholine (PtC), is exposed on the cell membrane. Detection of PC triggers the production of natural IgM antibodies and attracts phagocytes to remove the apoptotic cells (10).

Cell death and stress are hallmarks of rapidly growing cancer cells. We postulate that the cancer immune surveillance system may utilize natural IgM and natural IgM-producing B cells to recognize cancer cells under stress and apoptosis and rally danger signals to other immune cells. Supporting this notion, it has been reported that all human antibodies that bind tumor but not healthy tissue were germ-line-coded natural IgM antibodies produced by CD5^+^ human B-1a B cells (11, 12). These natural IgMs not only can recognize tumor, but can also induce apoptosis of cancer cells (13, 14). These studies suggest that humans possess a group of B cells that are similar to the natural IgM-producing murine B-1a B cells that generate antibodies as part of the immunosurveillance system that prevents and controls human cancer cell growth.

We have previously reported that approximately 50% of murine B-1a cells express PD-L2, a ligand for PD-1 (15). We termed these B-1a B cells L2pB1 cells. L2pB1 cells are distinct from other B cells in that they express self-recognizing, poly-reactive, and germ line-coded, natural IgM antibodies (16). In addition, L2pB1 cells express high levels of co-stimulation molecules, including PD-1, PD-L1, CD80, and CD86. As a result, L2pB1 cells are more potent antigen presenting cells (APC) than other B cells (16, 17). PD-L2 expression on L2pB1 cells does not seem to inhibit T cells and instead, we showed that L2pB1 cells strongly promote T cell proliferation and cytokine production (17). L2pB1 cells are the major source of self-reactive natural IgM antibodies in the serum, which are essential to immunosurveillance (17). In addition, L2pB1 cells secrete the regulatory cytokine IL-10 at higher levels than all other B cells tested, including Breg cells, at both baseline and activated states (18). We recently found that L2pB1 cells are able to phagocytize in a target-specific fashion, distinguishing them from traditional phagocytes such as macrophages and dendritic cells (19). This target specificity is likely contributed by the natural IgM antibodies which recognize stress-induced tumor cell membrane structures (20). For example, phosphatidylcholine (PtC)-binding B cells are contained mostly in the L2pB1 cell population (21). PtC and its head group, phosphorylcholine (PC), a major component of oxidized LDL (oxLDL), are exposed on cell surface during oxidative stress or cell death (21). In human, an anti-oxLDL natural IgM antibody (22) was shown to induce cancer cell lipoptosis (14). We have shown that B-1a cells selectively phagocytose PtC-decorated particles but not control particles whereas macrophages phagocytose both indiscriminately (19). These functions suggest that the L2pB1 cell population might contribute to the detection and removal of cancer cells through some new mechanisms that were not recognized before.

Here, we report the impact of the loss of L2pB1 cells in cancer immunity using a murine melanoma model. Our results suggest that L2pB1 cells play key roles in tumor recognition, cell death, and growth inhibition.

## Materials And Methods

### Mice

All procedures involving animal use were approved by the Boston University Medical Campus Institutional Animal Care and Use Committee (IACUC). Animals were cared for in compliance with the “Principles of Laboratory Animal Care” formulated by the National Society for Medical Research and the “Guide for the Care and Use of Laboratory Animals”. PD-L2–ZsGreen–TdTomato–diphtheria toxin receptor (PZTD) mice on a C57BL/6 background were generated and bred in the animal facility at Boston University School of Medicine as previously described (18). Briefly, these mice contain a diphtheria toxin (DT) receptor knock-in which allows for the inducible and specific ablation of L2pB1 cells without interfering with the development of macrophages or dendritic cells. PD-L2 expressing cells include L2pB1 cells, activated dendritic cells, and macrophages and are labeled with ZsGreen which is flanked by two LoxP sites. Crossing with a B cell–specific CD19-driven Cre recombinase transgenic mouse, the CD19-Cre^+/-^ PTZD^+/+^ mouse, will result in L2pB1 cells expressing TdTomato while macrophages and dendritic cells maintain ZsGreen expression. Female mice were used in most of the experiments unless specified as mixed gender. No sex-based differences were observed. All mice were cared for in an ABSL-2 room with standardized circadian cycles and diet. Sterile cages and equipment were used when handling mice at all times.

### L2pB1 Cell Depletion

CD19-Cre^+/-^ PTZD^+/+^ mice received one intraperitoneal injection of 25 ng/g body weight of Diphtheria Toxin (DT) (List Labs) every day for 4 days. One booster injection was given one week after the 4^th^ injection during the 16-25 day experiment. Depletion of L2pB1 cells was confirmed by flow cytometry (**Supplementary Figure S1**) as previously reported (18).

### Tumor Model

Murine melanoma (B16F10) cells were acquired from American Type Culture Collection (ATCC^®^ CRL-6475) and cultured in DMEM with 10% FBS. Each mouse received 0.5 × 10^6^ B16F10 cells *via* subcutaneous injection on the right flank. Tumor growth was monitored by palpation every two days until they could be monitored visually. Actual tumor size was calculated by tumor mass. At the point when surface necrosis of the tumor was observed or if a tumor exceeded 1.5, cm in diameter as measured by calipers, the experiment was terminated.

### MC38-TdT-C-7-C Cloning

The murine colon cancer cell line, MC38, was acquired from Dr. Yan Wu (Beth Israel Deaconess Medical Center) and was genetically engineered to have constitutive expression of TdTomato fluorescent protein and caspase 7-activatable expression of Cerulean fluorescent protein (CA-CerFP). CA-CerFP was constructed by linking the DEVD↓FQGP caspase 7-cleavable peptide sequence to a GFP sequence with the following codon replacement: F64L, S72A, Y66W, Y145A, and H148D (23). The resulting CA-CerFP gene was then synthesized (Genewiz) and cloned into the lentiviral vector pHAGE2-FullEF1a-ZsGreen-IRES-DTomatoW-T between NotI and BamHI sites (24). The lentiviral vector was packaged and transfected as previously described and resulted in a lentiviral insert that retains TdTomato but is without ZsGreen (25). Therefore, the modified MC38 cell line expresses the TdTomato red fluorescent protein, and the blue Cerulean fluorescent protein is expressed when caspase-7 is activated during cell death.

### B16F10-DsRed Cloning

The lentiviral vector, pHAGE2-FullEF1a-DsRedExpress-IRES-Puro-W was kindly donated by Dr. G. Mostoslavsky (Boston University School of Medicine) and packaged and transfected into the murine melanoma cell line B16F10 as previously described  (25). The resulting cell line constitutively expresses DsRed fluorescent protein.

### IVIS

Luciferase-expressing B16F10 cells were provided by Dr. Yan Wu (Beth Israel Deaconess) and subcloned (B16F10-Luc-R) in house. Each mouse received 0.5 × 10^6^ B16F10-Luc-R cells *via* subcutaneous injection as described above. To evaluate tumor size by the expression of luciferase, mice maintained under steady isoflurane anesthesia for several minutes were injected with 150 mg/kg luciferin (PerkinElmer, 122799) intraperitoneally. Anesthesia was maintained post-injection for the remainder of the experiment. To ensure a consistent signal and analysis, imaging was started at 15 minutes post luciferin injection and bioluminescent images were captured every 3 minutes using the IVIS^®^ Spectrum *in vivo* imaging system (PerkinElmer) until the signal started to diminish for all tumors (approximately 40 minutes after luciferin injection). Using the Xenogen Living Image 3.2 software, measurements of photon flux were used to generate a kinetic curve of luciferin uptake for each tumor. The peak bioluminescent signal for each tumor was used as an indicator of tumor viability on the day of measurement.

### Isolation of Tumor-Infiltrating Lymphocytes (TILs)

After dissection, tumors were minced, and tissue pieces were placed into DMEM containing collagenase type IV (low in trypsin; Worthington Bioscience, LS004188) and DNaseI (Alfa Aesar, J61061). Tumor tissue was digested for 45 minutes at 4° C with overhead rotation. Subsequently, 2 volumes of DPBS with 0.5% FBS were added, and EDTA was added to a final concentration of 2 mM. A syringe plunger was used to physically dissociate the remaining tissue pieces against a 70-μm filter and the cell solution was filtered again through a 70-μm filter. Cells were then washed once, and lymphocytes were isolated by standard Ficoll separation and used for flow cytometry.

### Immunohistochemistry

Tissues were dissected and fixed in 10% neutral buffered formalin (Fisher Scientific, SF99-4) for 24 hours at room temperature and processed in the Hypercenter XP (Thermo Fisher) using a standard dehydration protocol before paraffin embedding and microtome sectioning into 4 μm sections. Tissue sections were deparaffinized with xylene substitute (Thermo Fisher) before rehydration with gradients of alcohol. Heat-induced antigen retrieval was performed in Rodent Decloaker solution (Biocare Medical, RD913) in the Decloaking chamber (Biocare Medical). Non-specific interaction of primary antibodies with the tissue was blocked by Rodent block M (Biocare Medical, RBM961). Tissues were then stained with Rabbit anti-CD34 (Abcam, ab81289) at 1:3000 dilution in Da Vinci Green diluent (Biocare Medical, PD900) overnight at 4° C. The tissue section was stained with Rabbit-on-rodent-HRP polymer (Biocare Medical, RMR622). The slides were treated with Vector ImmPact^®^ VIP peroxidase substrate (Vector labs, SK-4605) for 10 minutes at room temperature. Methyl green (Vector labs, H-3402) was used to counterstain.

### Assessment of Angiogenesis

Depending on the size of the tumor, two to five 4 μm-thick sections, each of which was at least 100 μm from the subsequent section, were collected from each tumor. Anti-CD34 immunohistochemistry was performed on each tissue section and imaged at 100X magnification using the Keyence BZ-X700 microscope. CD34 quantification was performed using the ‘Hybrid cell count’ module of the Keyence Analysis. The 5 fields of images with the highest percentage of CD34^+^ area (angiogenic “hot spots”) (4) were selected from each section and used for quantification. CD34 expression from each tumor block was then averaged to get the final CD34^+^ area percentage of the tumor sample.

### Assessment of Lipoptosis

Cancer cells were cultured alone or with PCW cells for 72 hours. Naïve or LPS-activated PCW were compared. LPS was purchased from InvivoGen (Cat# tlrl-pb5lps). For activation, PCW were pre-treated with 1μg/ml LPS for 48 hours unless otherwise indicated. Cancer cells were then fixed with 4% PFA and stained with CD45-AF488 (BioLegend, Cat# 103122) as leukocyte marker, Oil Red O (ORO) (Sigma, confirm Cat#) for lipid stain and Hoechst 33342 (Invitrogen, Cat# R37605) as nuclear stain (**Supplementary Figures S2A, B**). Lipoptosis was quantified by measuring the average area of ORO staining in each cancer cell. Cancer cells were identified as CD45 negative cells. Cancer cell number per field was determined by counting Hoechst 33342 staining per field. Leukocytes were quantified by both areas of Hoechst 33342 staining and CD45-AF488 staining. ORO staining in cancer cells and lymphocytes were differentiated and counted respectively in the entire field. Average area of ORO stain per cancer cell was used as the final measure of lipoptosis quantification (**Supplementary Figure S3**).

### Flow Cytometry Analysis and Cell Sorting

The following antibodies were used to stain PCW cells: B220 PerCP-Cy5.5 (BioLegend, 103236), CD5 PE-Cy7 (BioLegend, 100622), CD19 BUV395 (BD Biosciences, 563557), IgM APC (Biolegend, 406509), PD-L2 BV421 (BD Biosciences, 564245), CD11b APC-Cy7 (BioLegend, 101226), Live/dead Ghost violet 510 (Tonbo Biosciences, 13-0870). For cells isolated from PZTD^+/+^ mice, ZsGreen fluorescent protein was analyzed using the same channel as FITC. For cells isolated from CD19-Cre^+/-^ PTZD^+/+^ mice, TdTomato fluorescent protein was analyzed using the same setting for TexasRed. To analyze TILs, CD45 PerCP-Cy5.5 (eBioscience, 45-0451-80), was added to the above panel replacing B220 for leukocyte gating. The stained samples were analyzed at the Boston University Medical Campus Flow Cytometry core facility using a BD LSRII flow cytometer and a Beckman Coulter MoFlo for cell sorting. Live leukocytes were gated by forward and side scatters, live/dead Ghost violet 510 and CD45 staining. B1 cells were gated as B220IntCD5Int population from the live leukocyte gate. L2pB1 cells were gated as PD-L2+IgM+ from the B1 cell gate. In the transgenic-knock in mice, L2pB1 cells were also identified by the expression of ZsGreen and TdTomato (**Supplementary Figure S1**).

### 2D B16F10 Cell Culture

Luciferase-expressing and TdTomato-expressing B16F10 cells were provided by Dr. Yan Wu (Beth Israel Deaconess Medical Center, Boston, MA) and modified as described above. Cells were cultured in Dulbecco’s Modified Eagle’s Medium with 10% fetal bovine serum, and 1% penicillin/streptomycin. The B16F10 luciferase-expressing cells were also maintained in media supplemented with 15 μg/mL puromycin. Experiments were performed within a week after thawing cells of passage 3 to 6.

### 3D Spheroid Culture and Quantification

B16F10 cell spheroids were created using the Cultrex Spheroid Formation ECM (SFM) (Trevigen, 3500-096-01) according to the manufacturer’s instructions. Briefly, 10,000 B16F10 cells were mixed in 50 µL of 1X SFM supplemented DMEM and transferred into each well of a 96-well, round bottom, ultra-low attachment (ULA) spheroid microplate. The plate was then centrifuged to center the cells followed by a 48-hour incubation period. 150 µL DMEM supplemented with SFM was carefully added to the top of each spheroid matrix droplet. After 4 additional days of incubation, the spheroids were used for inhibition assays.

3D tumor spheroids of the mouse colon cancer cell line MC38 were created using the hanging drop method (26). A single cell suspension of MC38 cells was prepared containing approximately 10,000 cells per 50 µL droplet. The droplets were placed on the underside of a 10 cm tissue culture plate lid and were held in place by surface tension, microgravity forces, and cell-cell adhesion when the lid was inverted. The tissue culture plate was kept in an incubator at 37°C with 5% CO_2_ and left undisturbed for 4-6 days to allow the cells to grow and form spheroids. PBS or media was added to the plate to prevent the droplets from drying out. One spheroid per well was transferred to a low adhesion 96-well plate in 100 μL of complete DMEM. Two hours after transfer, the spheroids were treated with various conditions and cultured for 8 to10 days.

### L2pB1 Hybridoma Clone Screening for Tumor Spheroid Binding

Twenty-eight L2pB1 cell hybridoma clones were obtained by fusion FACS-sorted L2pB1 cells with SP20 myeloma cells as previously reported (16). To test whether IgM antibodies secreted by these L2pB1 cells could recognize cancer cells, supernatant from each hybridoma clone was collected and verified for the presence of IgM using Rapid Mouse Antibody Isotyping XpressCard (ISO-M8a, Antagen Biosciences). In order to ensure that the spheroids were treated with comparable amounts of IgM-equivalent supernatant, an ELISA with IgM standard curve was performed on three dilutions of supernatant from each clone. The ELISA was prepared using goat anti-mouse Ig, human ads-UNLB (Cat# 1010-01, Southern Biotech) as a capture antibody and goat anti-mouse IgM, human ads-HRP (Cat# 1020-05, Southern Biotech) for detection. 1-Step ULTRA TMB-ELISA substrate solution (Cat # 34028 Thermo fisher) was added and the reaction was stopped with stop solution (Cat # SS04 Invitrogen). MC38-TdT-C-7-C cells were used to prepare spheroids using the previously described hanging drop method. The spheroids were transferred to a regular 96-well plate and incubated in 100μl of undiluted L2pB1 cell hybridoma supernatant at 37 °C for 3 hours followed by staining with anti-mouse IgM-AF488 secondary antibody (Invitrogen, A21042) for 30 min. The spheroids were imaged with the Keyence BZ-X700 microscope and 3D images were obtained using the z-stack capture module. Full focus z-stack images were then created using the Keyence Analysis software. To investigate the binding of IgM antibodies to regular mouse tissue, normal mouse embryonic fibroblasts (MEFs) (ATCC^®^ SCRC-1008) were used to prepare spheroids using the Cultrex Spheroid Formation ECM (Trevigen) and the hanging drop method. The MEF spheroids were stained with L2pB1 hybridoma supernatants and imaged as described above.

### Statistical Analysis

Statistical analysis was performed using GraphPad Prism 8 software. The ROUT method in Prism was used to identify outliers to be removed in each set of data points and the aggression level, defined as Q or False Discovery Rate (FDR), was set to 2%. Significant differences were evaluated by performing an unpaired, two tailed, t-test with Welsch’s correction (unequal variances confirmed by using F-test) and a confidence interval of 95%. P < 0.05 was considered a significant difference between compared groups.

## Results

### L2pB1 Cells Accumulate Inside Melanoma

**Figure 1:**
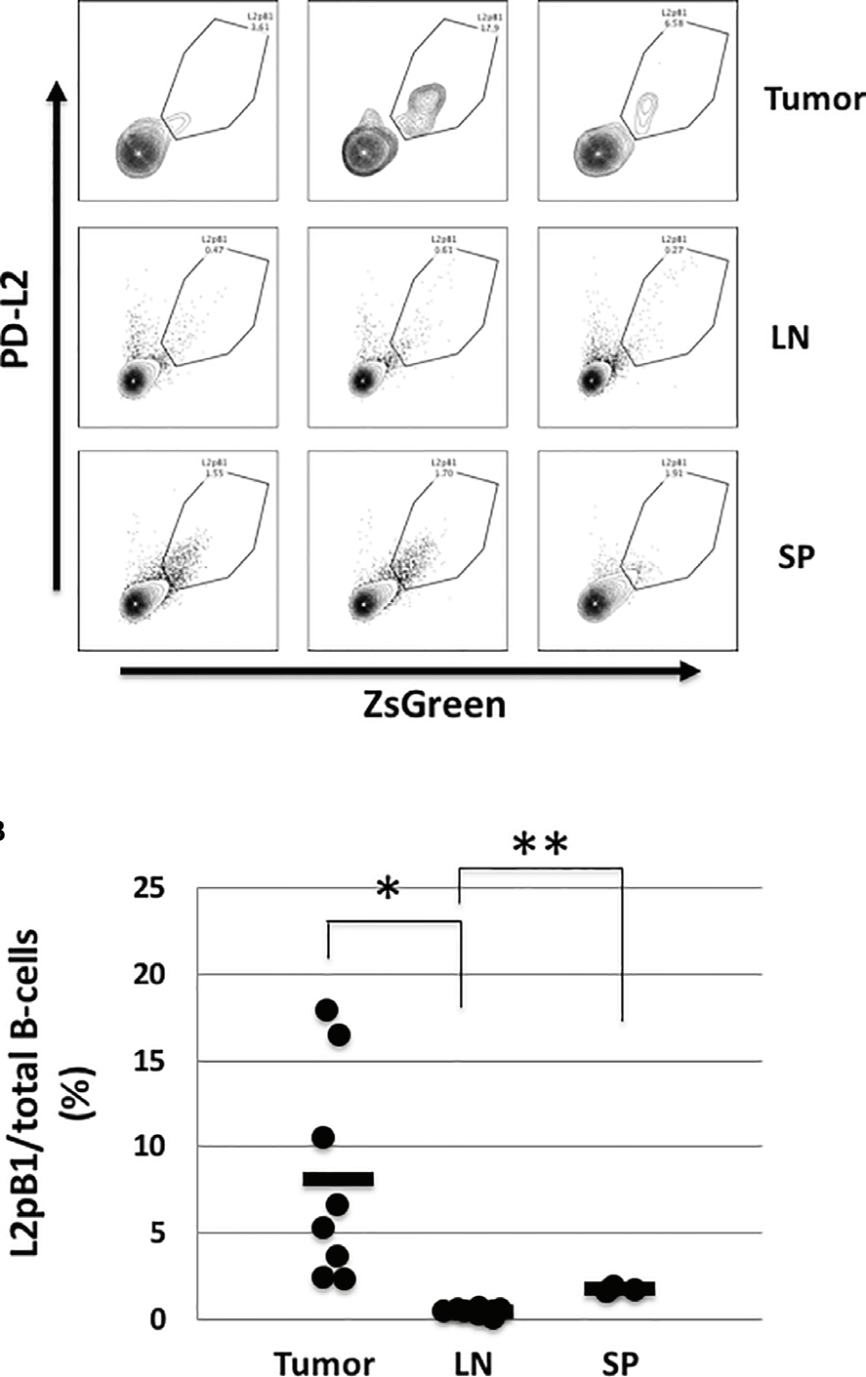
L2pB1 cells actively accumulate in tumors. Tumors were dissected on day 18 post injection. Lymphocytes were subjected to immunophenotyping by flow cytometry with fluorescently-labeled antibodies for CD45, CD3e, CD19, B220, IgM, PD-L2, and ZsGreen. **(A)** Representative histograms of PD-L2^+^ZsGreen^+^ L2pB1 cells in tumor, lymph nodes (LN), and spleen (SP). L2pB1 cells were identified as CD3^-^CD19^+^B220^+^IgM^+^PD-L2^+^ZsGreen^+^. **(B)** Percentage of L2pB1 cells from total tumor-infiltrating B cells as isolated from tumors (TIL), lymph nodes (LN), or spleen (SP). (*P = 0.01, **P = 0.02). Data are representative of three independent experiments.

Recent studies have shown that the presence of tumor-infiltrating lymphocytes (TILs), including B lymphocytes, is correlated with better prognosis (4, 5). However, the types of B cells making up the B-TIL population have not been fully elucidated. Since natural IgM (nIgM) has been reported as a distinguishing factor between tumor and normal tissue, we investigated whether nIgM-producing B cells are present inside tumors and play a direct role in anti-tumor immune response. We were particularly interested in PD-L2-expressing B-1 B cells (L2pB1 cells) due to their poly-self-reactive nature. We previously generated and validated the use of a transgenic animal model where L2pB1 cells can be tracked by a fluorescent protein, ZsGreen. The CD19-Cre-PZTD model also allows L2pB1 cells to be inducibly depleted by an intraperitoneal injection of Diphtheria Toxin (DT). Using this model, we induced melanoma by subcutaneous injection of B16F10 cells, and collected tumors on day 18 post-inoculation. TILs were isolated and subjected to flow cytometry. L2pB1 cells were identified as CD19^+^PD-L2^+^ZsGreen^+^IgM^+^ cells (**Figure 1A**). The percentages of L2pB1 cells among total B cells in tumor, inguinal lymph nodes, and spleens were assessed (**Figure 1B**). We found <1% L2pB1 cells in lymph nodes and spleen from tumor-inoculated mice, a finding similar to previous reports showing that L2pB1 cells account for less than 1% of total B cells in lymph nodes, spleen, and blood from healthy mice. On the other hand, L2pB1 cells accounted for up to 18% of the total number of B cells in tumors, with a mean around 8%. Therefore, TILs are highly enriched with L2pB1 cells compared to other lymphoid organs in B16F10 induced melanoma-bearing mice.

### Depletion of L2pB1 Cells *In Vivo* Resulted in Increased Tumor Mass and Angiogenesis

**Figure 2:**
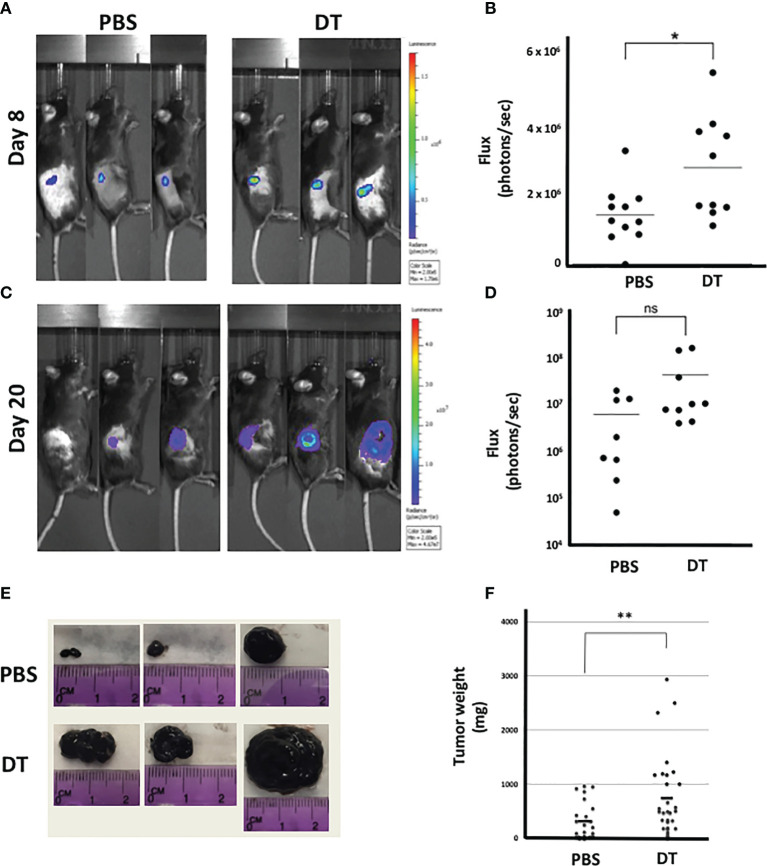
Depletion of L2pB1 cells increases tumor mass. CD19-Cre-PZTD transgenic mice received daily intraperitoneal injection of PBS or diphtheria toxin (DT) for 4 days to deplete L2pB1 cells prior to subcutaneous inoculation with either luciferase-expressing (Luc-R) or WT B16F10 melanoma cells. Representative peak bioluminescent signal acquired by IVIS on **(A)** day 8 and **(C)** day 20 (n=9 PBS; n=10 DT). Bioluminescent flux quantification for **(B)** day 8 post-inoculation and **(D)** day 20 post-inoculation. **(E)** Gross observation of B16F10 melanoma tumor growth in representative PBS and DT-injected mice. **(F)** Comparison of end-point B16F10 tumor weights between PBS-injected (n=24) and DT-injected (n=29) mice. ns, not statistically significant; *P = 0.0212, **P = 0.0082. Data shown lare pooled from at least three independent experiments.

To assess the role of L2pB1 cells *in vivo*, we again utilized the CD19-Cre-PZTD model (18). B16F10 melanoma cells were inoculated by subcutaneous injection after 4 consecutive daily intraperitoneal injections of DT to ensure 70-90% depletion of L2pB1 cells. A luciferase-expressing B16F10-Luc-R cell line was used for evaluation by *in vivo* imaging. Starting at day 3 post-inoculation, tumor growth was assessed, and tumor size was evaluated by measuring the peak total flux of bioluminescent signal of each tumor (**Supplementary Figure S1**). There was no observable difference between the groups on day 3 since the tumors were not yet well established (data not shown). However, on day 8, there was a statistically significant increase of the total bioluminescent signal in the DT-treated mice compared to PBS (**Figures 2A, B** **)**. On day 20, prior to terminating the experiment, the total bioluminescent signal trended towards an increase in the DT-treated compared to PBS-treated mice (**Figures 2C, D** **)**. Necrosis was observed in many large tumors on day 20, corresponding to the loss of bioluminescent signal (**Figure 2C**).

On day 21-post tumor inoculation, the experiment was ended and tumors were dissected and weighed (**Figures 2E, F** **)**. In contrast to the IVIS data, the terminal tumor weight was significantly increased in the DT-treated mice compared to PBS (**Figure 2F**). Due to the reduction of the bioluminescent signal in necrotic tumors on day 20, the significant change in tumor weight may reflect a more accurate difference between the two experimental groups.

**Figure 3:**
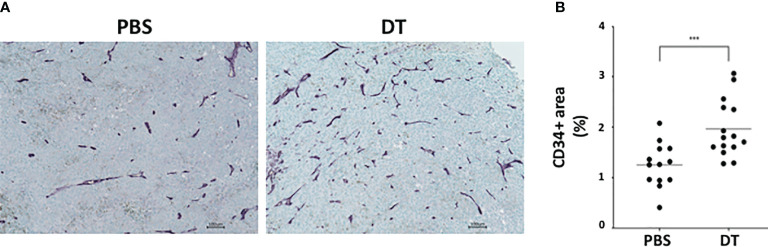
Depletion of L2pB1 cells increases angiogenesis in melanoma. CD34 expression in **(A)** PBS-injected control and DT-injected B16F10 melanoma tumors assessed by immunohistochemistry. **(B)** Angiogenesis was quantified as the ratio of total CD34-positive areas (purple) to the total tumor tissue area. ***P = 0.0009. Data are pooled from three independent experiments.

As blood vessel development plays a critical role in tumor growth, we assessed the extent of angiogenesis within the groups. More blood vessels were observed around the larger tumors in the DT-treated mice compared to the PBS-treated mice. To determine the extent of angiogenesis in the tumors of L2pB1 cell-depleted mice, paraffin-embedded samples were assessed by immunohistochemistry for CD34 expression (**Figure 3A**). Angiogenesis was quantified by measuring the CD34^+^ areas in “hot spots” (i.e*.*, the five highest positive equal-size fields of each tissue section). CD34^+^ vessel area was expressed as a percentage of the total area (**Figure 3B**). Our results demonstrate significantly increased CD34^+^ areas, suggesting increased tumor angiogenesis and confirming tumor growth in the absence of L2pB1 cells.

### L2pB1 Cells Contribute to Cancer Cell Growth Inhibition and Apoptosis

**Figure 4:**
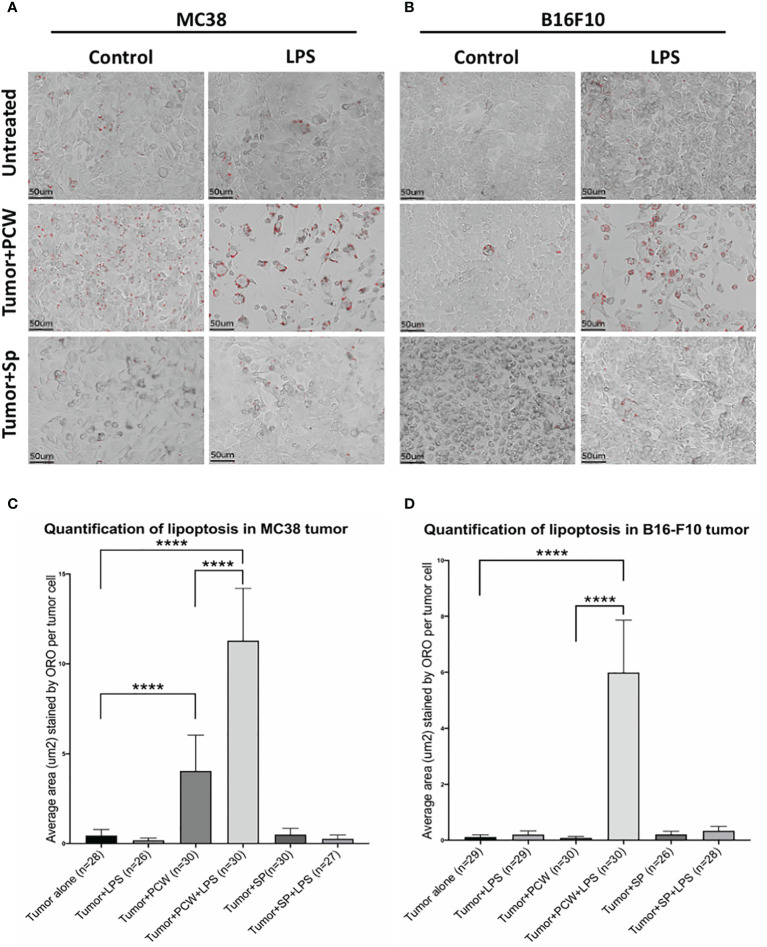
L2pB1 cells are essential for PCW-induced lipoptosis of melanoma cells. **(A)** MC38 colon cancer cells and **(B)** B16F10 melanoma cells were cultured alone, or co-cultured with PCW cells or splenocytes for 72 hours in the presence and absence of LPS. Oil Red O staining was performed at 72 hours. Scale bar (red) = 50μm. Representative images from triplicate wells were taken at 400x magnification. **(C)** Oil Red O intensity analysis of MC38 cells co-culturing with PCW cells in the presence or absence of LPS. There is significant difference between cancer cell alone and cancer cells in the presence of PCW cells (****p value <0.0001). Stimulation of PCW cells with LPS during co-culture induced significantly greater amount of lipoptosis as compared to that in the absence of LPS (****p value <0.0001). **(D)** Oil Red O intensity analysis of B16F10 cells lipoptosis. The intensity was significantly increased upon co-culture with PCW cells in the presence of LPS (****p value <0.0001).

One way in which natural IgM induces tumor cell apoptosis is through the poly-reactive feature that binds extracellular cholesterols and cancer cell membrane phospholipids at the same time. Such binding results in cholesterol internalization and cell death by overloading of the lipids, known as lipoptosis (14). Approximately 10~30% L2pB1 cells are poly-reactive to lipids and can phagocytose PtC-covered liposomes (19). To determine if L2pB1 cells are essential for peritoneal cavity washout (PCW)-induced cancer cell growth inhibition and lipoptosis, we performed a side-by-side comparison of MC38 colon cancer cells (**Figure 4A**) and B16F10 melanoma cell (**Figure 4B**) cultured with PCW, L2pB1-depleted PCW cells (PCW-DT), or splenocytes (**Supplementary Figure S2**). Since L2pB1 cells can be activated by LPS, we used LPS treatment to determine if the effect on lipoptosis could be enhanced.

Quantification of Oil Red O staining (methods detailed in **Supplementary Figure S3** **)** to determine the extent of lipoptosis in the tumor cells demonstrated significantly increased lipoptosis in MC38 (**Figure 4C**) and B16F10 (**Figure 4D**) cells in the presence of PCW. As tumor cells do not typically accumulate lipid droplets, the presence of Oil Red O stained lipid droplets and less dense cell populations suggests a role of L2pB1 in cancer cell inhibition. Our results reveal that PCW, but not splenocytes, can induce lipoptosis of two cancer cell types and this effect is enhanced by LPS stimulation.

**Figure 5:**
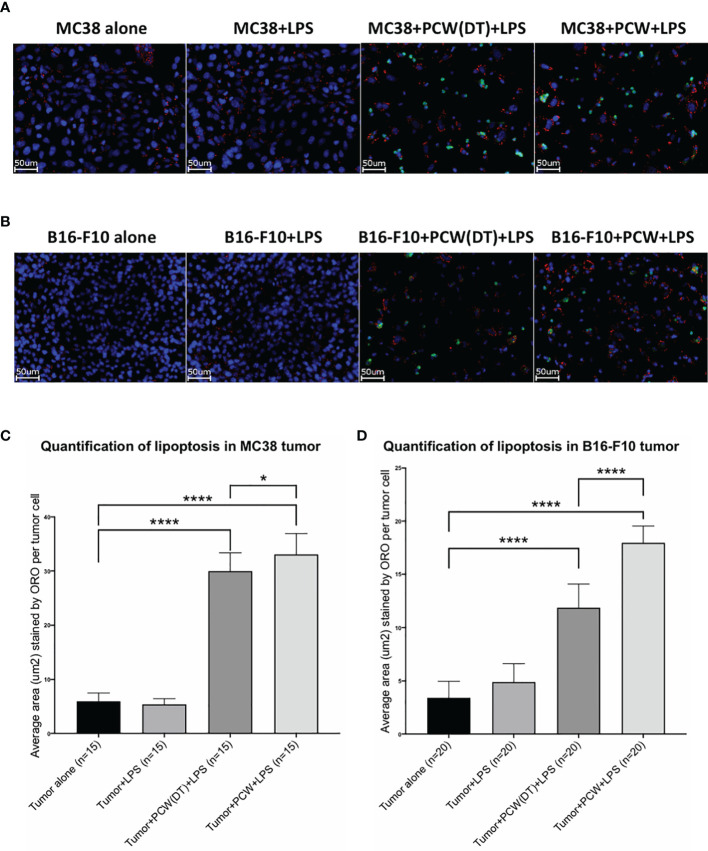
Depletion of L2pB1 cells reduces lipoptosis of both MC38 and B16F10 tumor cells. **(A)** MC38 colon cancer cells and **(B)** B16F10 melanoma cells were cultured alone or co-cultured with PCW cells from saline or DT treated mice for 72 hours, in the presence of LPS. Cells were stained with Hoechst 33342 (nuclei), CD45-AF488 (leukocytes) and Oil Red O at 72 hours. Representative images from triplicate wells were taken at 400x magnification. Scale bar (white) = 50μm. **(C, D)** Lipoptosis was quantified as average area of Oil Red O staining per tumor cell (**Supplemental Figure 3**). MC38 and B16F10 tumor cells treated with L2pB1-depleted PCW cells showed significant reduction of lipoptosis as compared to wild type PCW cells, ****p < 0.0001 and *p = 0.0265.

To further investigate the importance of L2pB1 cells for lipoptosis, PCW cells were obtained from DT-treated, L2pB1-depleted mice and compared to that of PBS-treated control mice (**Figure 5**). We demonstrate that WT PCW induced significantly more lipoptosis than L2pB1-depleted PCW, in both MC38 (**Figures 5A, C** **)** and B16F10 (**Figures 5B, D** **)** cancer cells, indicating L2pB1 cells in PCW play an important role in lipoptosis induction. Furthermore, splenocytes did not induce lipoptosis in cancer cells under these conditions (**Figures 5A, B** **)**.

### L2pB1 Cells Inhibit the Growth of 3D Tumor Spheroids

**Figure 6:**
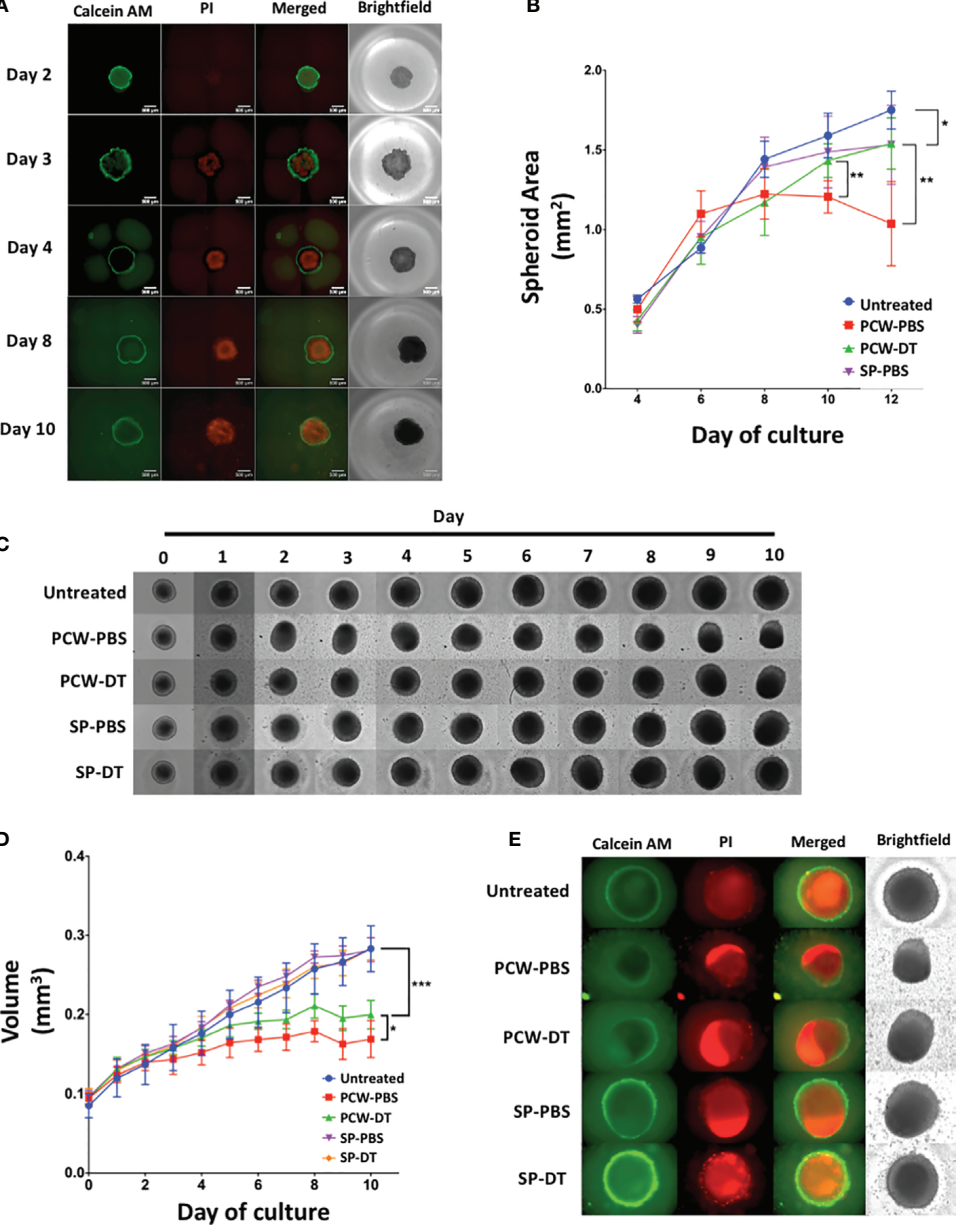
L2pB1 cells inhibit tumor spheroid growth *in vitro*. **(A)** Calcein AM (CAM) and Propidium Iodide (PI) staining of B16F10 tumor spheroids (n=8). **(B)** Time course of spheroid size after treatment with various cell types (*P < 0.05; **P < 0.01). **(C)** Representative MC38 spheroids from each treatment group (n=8) over a 10-day co-culture period: untreated, PCW-PBS, PCW-DT, SP-PBS or SP-DT. **(D)** Time course of MC38 spheroid growth under different treatments. (*P < 0.05 (PCW-PBS *vs.* PCW-DT; ***P < 0.0001 [control *vs.* PCW-PBS)]. **(E)** Representative IF images on co-cultured MC38 spheroids at day 10. Data are representative of at least three independent experiments.

As shown in **Figure 1**, L2pB1 cells infiltrate and actively accumulate inside solid tumors *in vivo*. In addition, L2pB1 cells were required for the induction of tumor cell lipoptosis in 2D culture (**Figures 4**, **5**). To test whether L2pB1 cells can inhibit solid tumor growth, B16F10 tumor cells were cultured on low adhesion plates and allowed to form solid tumor spheroids for 4 days before adding PCW-PBS or PCW-DT cells (**Figure 6A**). On day 3, the B16F10 tumor spheroids start to show a PI-positive necrotic core, while Calcein AM-positive live cells formed a typical green ring outside the necrotic core (**Figure 6A**). This showed the growing phases of the spheroids that mimic tumor growth. Measurements of spheroid size every two days revealed that co-culture with PCW-PBS cells completely arrested spheroid growth around day 6, while spheroid growth continued uninhibited in the presence of L2pB1 cell-depleted PCW or splenocytes (**Figure 6B**). Similar, and statistically significant, patterns of inhibition were observed using spheroids obtained from MC38 colon cancer tumor cells (**Figure 6C**). PCW cells from PBS injected mice (PCW-PBS) show robust inhibition of spheroid growth after day 4, whereas tumors continue growing when cultured with PCW from L2pB1 cell-depleted mice (PCW-DT) (**Figure 6D**). Co-culture with splenocytes from both PBS and DT groups had no effect (**Figure 6D**). To investigate whether the tumor spheroid growth inhibition is associated with increased cell death, we assessed the size of the necrotic cores and observed that co-culture with L2pB1 cell-depleted PCW resulted in a larger overall spheroid size and observable necrotic core that is stained positive with PI (**Figure 6E**).

### Monoclonal IgM Secreted by L2pB1 Cell-Derived Hybridoma Cells Specifically Bind Tumor Spheroids but Not Embryonic Fibroblasts

**Figure 7:**
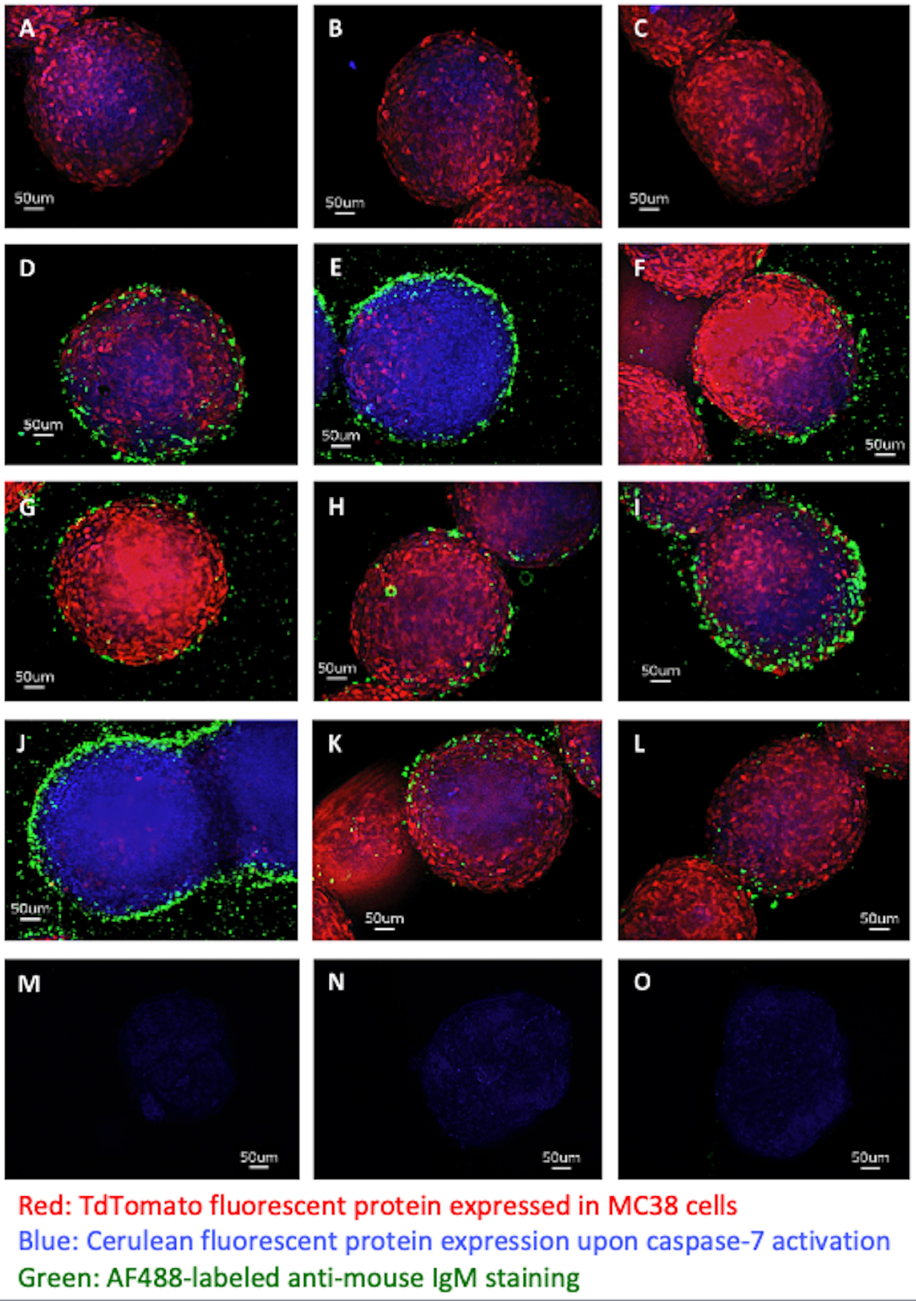
Monoclonal IgM antibodies from L2pB1-derived hybridoma specifically recognize tumor spheroids. 3D tumor spheroids made with MC38 cells (MC38-TdT-C-7-C) express TdTomato when alive and, depending on the expression level of Cerulean, display purple or blue upon cell death induction were incubated with monoclonal IgM antibodies derived from L2pB1 cell hybridoma clones and imaged at 400X magnification: **(A)** MC38 tumor spheroids stained with anti-mouse IgM-AF488 secondary antibody alone as negative control for non-specific binding; **(B, C)** MC38 spheroids stained with IgM antibodies from two negative control clones that do not recognize tumor; **(D–L)** MC38 spheroids stained by monoclonal IgM antibodies from 9 hybridoma clones that showed positive staining. **(M–O)** Spheroids prepared using normal mouse embryonic fibroblast (MEFs) cells were incubated with the supernatants of each of the L2pB1 hybridoma clones and imaged at 400X magnification. Scale bars for all images represent 50uM. Results are representative of at least three independent experiments.

Twenty-eight hybridoma clones derived from L2pB1 cells have been previously reported to have poly-reactivity to various self-antigens (**Supplementary Table S1**) (16). One explanation of why a single IgM can recognize so many different antigens at the same time is that these IgM might recognize some common non-amino acid epitopes of an antigen, such as lipids, carbohydrates, and DNA and RNA structures. To test if these L2pB1 cell-derived monoclonal IgM antibodies specifically recognize tumor cells but not normal, healthy growing cells, we tested the binding of L2pB1 cell-derived IgM antibodies on MC38 tumor spheroids or control MEF spheroids. The secreted IgM from 9 of the 28 L2pB1 cell hybridoma clones specifically bound and induced cell death of the MC38 tumor spheroids (**Figures 7A** **–L**), while none recognized MEFs (**Figures 7M–O** and **Supplementary Figure S4**). This further implicates the involvement of L2pB1 cells in tumor-specific immune response.

## Discussion

### The Effects of L2pB1 Cell Depletion on Tumor Growth

To evaluate the impact of L2pB1 cells in immunosurveillance *in vivo*, we examined the presence of L2pB1 cells in tumor-infiltrating lymphocytes (TIL). We found that L2pB1 cells actively accumulated inside melanoma as compared to lymph nodes, spleen, and peripheral blood. When we depleted L2pB1 cells using our previously generated mouse model that allows for DT-induced L2pB1 cell-specific depletion *in vivo* (18), we observed increased tumor mass and angiogenesis in the L2pB1 cell-depleted mice. Even though DT injection is local, the depletion of the L2pB1 cells is systemic (**Supplementary Figure S5**). Therefore, any circulating L2pB1 cells are likely depleted and cannot infiltrate into the tumor. These findings are highly suggestive of a role for the L2pB1 B cell subpopulation in cancer immunosurveillance and prompts understanding of the mechanism behind L2pB1 cell coordination with other immune cells.

PD-L2 has been reported to be expressed on subpopulations of memory B cells as well. Therefore, our data does not exclude the possibility that DT-mediated depletion of PD-L2 positive B cells other than L2pB1 cells might also contribute to the increase of tumor size. However, according to (Nat Immunol 2014), IgM+ memory B cells are actually PD-L2 negative, whereas PD-L2+ memory B cells are IgG+ B cells. Even though our focus is IgM+ B-1 B cells, further investigation is needed to exclude the involvement of PD-L2+IgG memory B cells. In naïve mice, we did not observe ZsGreen positive nor TdTomato positive B-2 B cells. Therefore, when we injected DT into naïve mice, it is unlikely that PD-L2+B cells other than B-1 cells were affected as much as L2pB1 cells. However, we do not exclude the possibility that in late stage of tumor development, many cell types might be activated upon metastasis. Therefore, IgM negative B cells, including PD-L2+IgG memory B cells, might expand and also be involved.

We investigated the interaction between L2pB1 cell-rich peritoneal cells and B16F10 melanoma cells. We found that melanoma cells undergo lipoptosis in the presence of L2pB1 cell-rich peritoneal cells but such lipoptosis was significantly diminished when L2pB1 cells were depleted. The depletion ranged from 70% to 90%, which aligns with the increased tumor sensitivity to DT compared to normal cells demonstrated by Buzzi et al. in Ehrlich tumors (27). Among rodents, guinea pigs are most sensitive and mice are least sensitive to DT. Cell resistance to DT is conceivably linked to the degree of macromolecular uptake (through pinocytosis, if DTR is not involved due to species differences) and to the cellular factors of self-defense against the action of microbial toxins. In this regard, neoplastic cells are more sensitive to DT than normal cells. Therefore if DT contributed any effects on tumor growth, it will shrink the tumor. However, in our DT treatment, B16 tumors grew larger, which further suggests a suppressive role of L2pB1 cells.

We also examined 3D tumor spheroid growth and observed similar tumor growth inhibition in both B16F10 melanoma and MC38 colon cancer tumor spheroids. In addition, as cell line behavior and sensitivity to lipoptosis and other PCW-mediated inhibition could vary, the apparent moderate but significant effect of removing L2pB1 cells from PCW on the growth of MC38 spheroids compared to B16F10 spheroids suggests that MC38 and B16F10 have different sensitivities to the growth inhibition and lipoptosis triggered by L2pB1. L2pB1 cell-derived monoclonal IgM antibodies also specifically bind MC38 tumor spheroids. Therefore, while lipoptosis may be playing a role, we do not rule out the potential contribution of coordinated interactions of different cell types for other tumor destructive mechanisms.

### Potential Mechanisms of L2pB1 Cell-Mediated Tumor Growth Inhibition

Our analysis of L2pB1 cells suggests that this B cell subpopulation plays important roles in recognizing and controlling tumor cell growth. The depletion of L2pB1 cells results in greater tumor growth, further suggesting a suppressive role. We also demonstrate that specific recognition of tumor cells is possible through the poly-reactive function of the IgM antibodies produced by L2pB1 cells. These antibodies recognize large, conserved, non-amino acid epitopes with repetitive structures, including lipids and carbohydrates, DNA and RNA (12, 28–30) which are only displayed on stressed tumor cells or are displayed in a much larger quantity compared to normal cells. The low affinity but high avidity of these IgM antibodies might explain the preferential binding to tumor cells with higher amounts of these repetitive molecules in stressed or dying tumor cells. In addition, while our data suggest that L2pB1-derived IgM not only bind tumors but also induce apoptosis, we do not exclude the possibility that some IgM can bind tumor cells without inducing apoptosis and may potentially be linked to the strength with which IgM binds to its target. These ideas are supported by reports showing these natural IgM antibodies play roles in regulating immune homeostasis (10).

In this report, we demonstrate that L2pB1 cells, a subpopulation of B-1B cell, inhibit tumor growth both *in vivo* and *in vitro*, providing promising information about this unique B cell population. The contribution of L2pB1 cells to cancer immunosurveillance may not be limited to the natural IgM. As previously reported, L2pB1 cells are potent antigen presenting cells (16, 17). In addition, we recently reported that L2pB1 cells could not only phagocytize, but also constitutively express the anti-inflammatory cytokine IL-10, more than any other types of B cells (18). This suggests that in addition to recognizing and removing cancer cells and presenting tumor antigen to T cells, L2pB1 cells may contribute to limiting tumor metastases by regulating inflammation through PD-L2 and IL-10 expression.

Our study supports the idea that B-1 cells play an anti-cancer role, corroborating evidence by Azevedo *et al.* which reports that B-1 cells obtained from a mouse model of Erlich tumor can protect a recipient mouse from the same cancer (31). In addition, a recent study using a murine peritoneal carcinomatosis model showed that B-1 cells and their IgMs are required for the anti-cancer response induced by the combination of toll-like receptor and C-type lectin receptor agonists (32). In this study, Haro *et al.* reported that B-1a cells, but not splenic or peritoneal B-2 cells, are able to reconstitute anti-tumor effects and that B-1 cell-derived IgM and complement are essential. Collectively, there is sufficient evidence of the important role of B-1 cells in the growth progression of several solid tumors, potentially indicating a novel therapeutic approach.

Although PD-L2 expression is a hallmark of L2pB1 cells and PD-L2 is a ligand for PD-1, it is reasonable to question whether PD-L2 on L2pB1 cells may contribute to immune suppression inside the tumor. It is important to note that PD-1 is expressed on both pro- and anti-inflammatory cells. Through the use of our unique mouse model, we can dissect the effects of PDL-2-expressing cells on immune infiltration in tumors. However, the net outcome of the interaction of PD-L2 on L2pB1 cells with PD-1 expressed on various immune cells inside the tumor microenvironment cannot be easily predicted and requires additional research. Furthermore, it has been reported that PD-L1 and PD-L2 might bind to receptors other than PD-1 (33). Therefore, at this stage, while we cannot draw definitive conclusions on the net contribution of PD-L2 expressed on L2pB1 cells, it is reasonable to speculate that L2pB1 cells facilitate lipoptosis. The modification of other phagocytes and T cells is certainly a consideration and future experiments could address this issue by using sorted L2pB1 cells and PDL-2^-/-^ B-1a (L2nB1) cells for adoptive transfer to Rag2^-/-^ mice or secretory Ig-deficient mice.

It is possible that the contribution of PD-L2 on L2pB1 cells could involve self-regulation and the regulation of other immune cells. We have reported that certain danger-associated signals, such as LPS and CpG or cross-linking by CD40L and surface IgM, could downregulate PD-L2 expression on L2pB1 cells (21). In terms of the role of PD-L2 on self-regulating feedback, it was recently reported that PD-L2 regulates the expression of anti-PC IgM by L2pB1 cells through an IL-5-dependent mechanism (34). Of further interest, it has been demonstrated that PD-L2 could differentially inhibit exhausted CD8^+^ T cells and Tregs in the tumor while sparing fresh CD8^+^ T cells (35). Thus, it is possible that L2pB1 cells may reverse the immunosuppressive tumor microenvironment by regulating T cell populations through PD-L2 and PD-1 signaling. Furthermore, while PD-L2 is also expressed on other types of B cells, including memory B cells, GCs, and activated B2 B cells, it is unlikely that the population described in our study are memory cells or IgG^+^ B cells since it has been reported that IgM^+^ memory B cells are PD-L2 negative (38). Furthermore, a recent *in vivo* study suggested that B-1 cells are also capable of expressing granulocyte-macrophage colony-stimulating factor (GM-CSF) upon activation (36). The anti-cancer effect of GM-CSF is well documented (37); however, the efficiency of administering GM-CSF in clinical treatment is limited and hard to control. It is reasonable to postulate that L2pB1 cells may produce GM-CSF upon activation and regulate the tumor microenvironment by adjusting local cytokines levels.

### Future Investigation of L2pB1 Cells in Tumor Immunology

Our data demonstrate that L2pB1 cells are critical for tumor suppression. L2pB1 cells are likely a true regulatory cell type, demonstrating plasticity, versatility, and the ability to adapt to their microenvironment. We believe that both soluble (e.g. secreted IgM, cytokines, and chemokines) and cell-cell contact (BCR, co-stimulatory or co-inhibitory molecules) are involved *in vivo*. Future experiments using blocking reagents in combination with transwell assays are needed to dissect each element involved.

Regulatory immune cells should not be confused with suppressive cells. Regulatory cells can be both stimulatory and suppressive depending on the microenvironment and physiological interaction with different cell types. A snapshot experimental system that only catches one state of the regulatory cells often generates controversial data due to the plasticity of the regulatory cell and the diversity of experimental systems. L2pB1 cells are a good example of such regulatory immune cells because they express both positive and negative stimulatory molecules. For example, L2pB1 cells express many pairs of molecules that have opposite regulatory functions. These include Fas and FasL, IL-6 and IL-10, and PD-1 and PD-L1/L2. Taken together, our data provide evidence of new protective roles the L2pB1 cells may play in the tumor microenvironment.

Polyreactive IgM derived from L2pB1 cells is a key player for cancer cell recognition, tumor growth inhibition, cancer cell death induction and removal. *In vitro* binding characteristics (**Supplementary Table S1**) may not reflect the actual *in vivo* binding. A real-time *in vivo* investigation of the binding of L2pB1-derived IgM and tumors will reveal critical information.

Even though our data suggests that L2pB1 cells are likely contribute to tumor growth inhibition, we do not exclude the possibility that under certain circumstances, immune cells can be hijacked by tumors that are evolved to utilize certain immune functions for growth advantage. Further investigation of the balance of suppression and activation of immune response during the interaction between L2pB1 cells and tumor cells within the tumor microenvironment will be critical for therapeutic consideration.

### Summary

In summary, our study has delineated a potential role of L2pB1 cells in cancer immunology. L2pB1 cells could contribute significantly to cancer immunosurveillance and the control of established cancers through poly-reactivity of tumor-recognizing nIgM, cell death-induction, PtC-specific phagocytosis, potent antigen presentation, and balanced expression of pro- and anti-inflammatory cytokines. Reduction or depletion of L2pB1 cells poses a risk of cancer cell accumulation. Most importantly, due to the nature of their tumor pattern recognition, L2pB1 cells and associated nIgM may reduce drug resistance due to tumor heterogeneity. While our study focuses on the known functions of L2pB1 cells, this does not exclude the possibility that the anti-tumor effects we saw are the results of a combined effort of L2pB1 cells and other immune cells. Further studies are needed to determine the potential therapeutic benefit of boosting L2pB1 cells for cancer treatment.

## Data Availability Statement

The original contributions presented in the study are included in the article/**Supplementary Material**. Further inquiries can be directed to the corresponding author.

## Ethics Statement

The animal study was reviewed and approved by Boston University Medical Campus Institutional Animal Care and Use Committee.

## Author Contributions

Conception and design: XZ. Development of methodology: VS, AB, RN, WG, and XZ. Acquisition of data: VS, AB, CM, HB, HV, AR, EL-B, SC, VC, JG, AK, TL, TV, and XZ. Analysis and interpretation of data: XZ, VS, and AB. Writing, review, and revision of the manuscript: XZ, VS, EL-B, CM, and WG. All authors contributed to the article and approved the submitted version.

## Funding

This study was supported by National Cancer Institute through Grant Number R21 CA205415, by National Institute of Diabetes and Digestive and Kidney Diseases through Grant Number 1R56DK103894-01A1, and by the National Center for Advancing Translational Sciences, National Institutes of Health, through BU-CTSI Grant Number 1UL1TR001430.

## Conflict of Interest

The authors declare that the research was conducted in the absence of any commercial or financial relationships that could be construed as a potential conflict of interest.

## Publisher’s Note

All claims expressed in this article are solely those of the authors and do not necessarily represent those of their affiliated organizations, or those of the publisher, the editors and the reviewers. Any product that may be evaluated in this article, or claim that may be made by its manufacturer, is not guaranteed or endorsed by the publisher.
