# Supplementary material for: L2pB1 Cells Contribute to Tumor Growth Inhibition
Source: Front Immunol. 2021 Sep 23;12:722451. doi: 10.3389/fimmu.2021.722451 (PMC8495424; doi:10.3389/fimmu.2021.722451)
Supplement: Supplementary file 1 [file DataSheet_1.pdf]

Supplementary Material

L2pB1 cells contribute to tumor growth inhibition

1 Supplementary Data

Figure S1. Depletion of L2pB1 cells in the peritoneal cavity of tumor-bearing mice

Figure S2. Individual fluorescence channels for representative images of tumor cells cultured under various conditions

Figure S3. LPS enhances lipoptosis of both MC38 and B16F10 cells

Figure S4. Monoclonal IgM antibodies from L2pB1-derived hybridoma do not recognize MEF spheroids.

Figure S5. Systemic depletion of L2pB1 cells

Table S1. Polyreactivity of L2pB1 hybridoma clones tested with different assays

1.1 Supplementary Figures

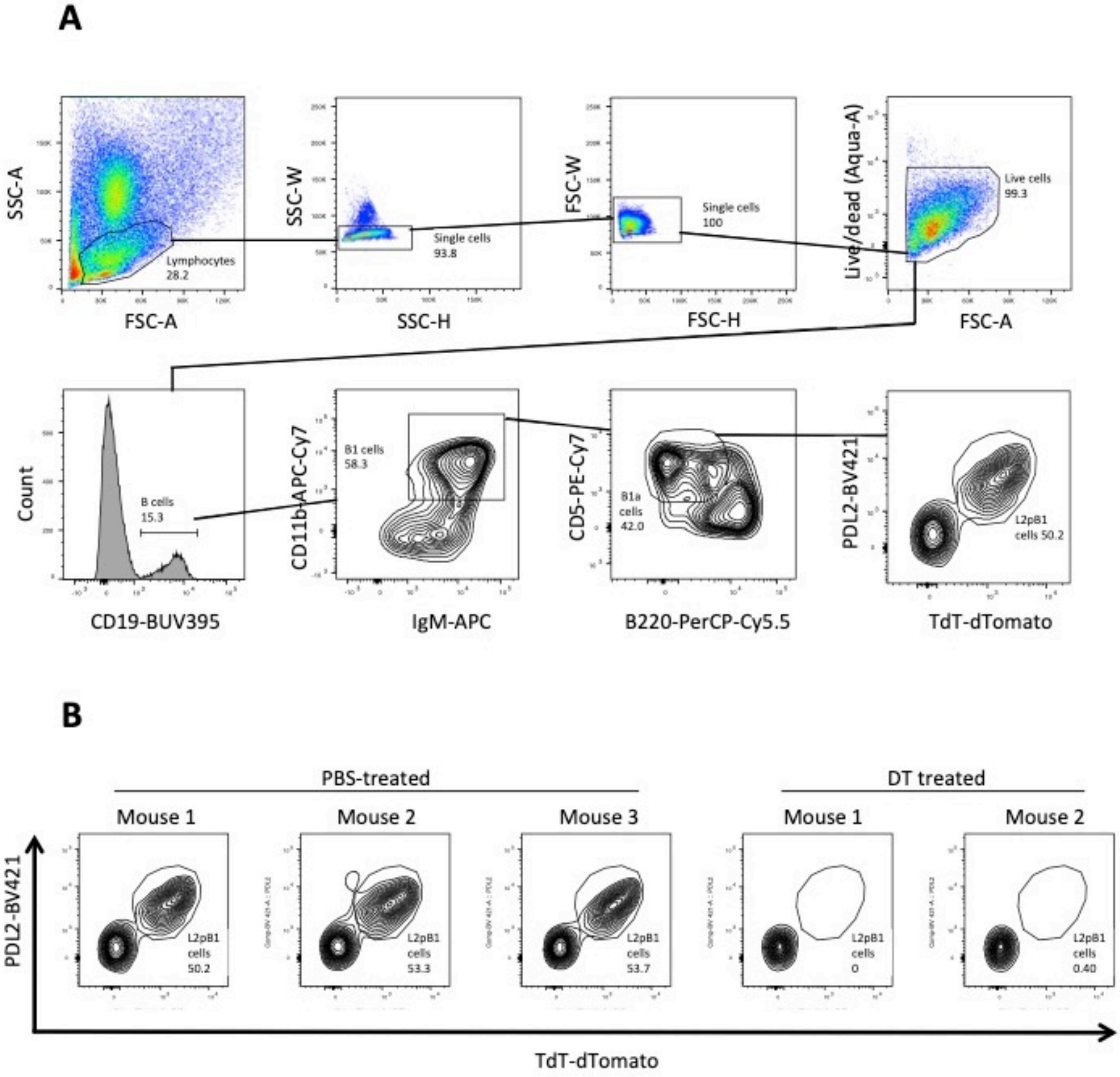

Figure S1. Depletion of L2pB1 cells in the peritoneal of tumor-bearing mice

C19-Cre+/- PZTD+/+ mice were injected intraperitoneally with DT or PBS 4 days before tumor inoculation. Peritoneal washout was obtained on day 18. (A) Flow cytometry gating strategy is shown. L2pB1 cells were identified as CD19+IgM+CD5+B220lowTdT+PDL2+. (B) L2pB1 cell populations as CD19+IgM+CD5+B220lowTdT+PDL2+ in the peritoneal cavity of mice treated with PBS or DT.

A

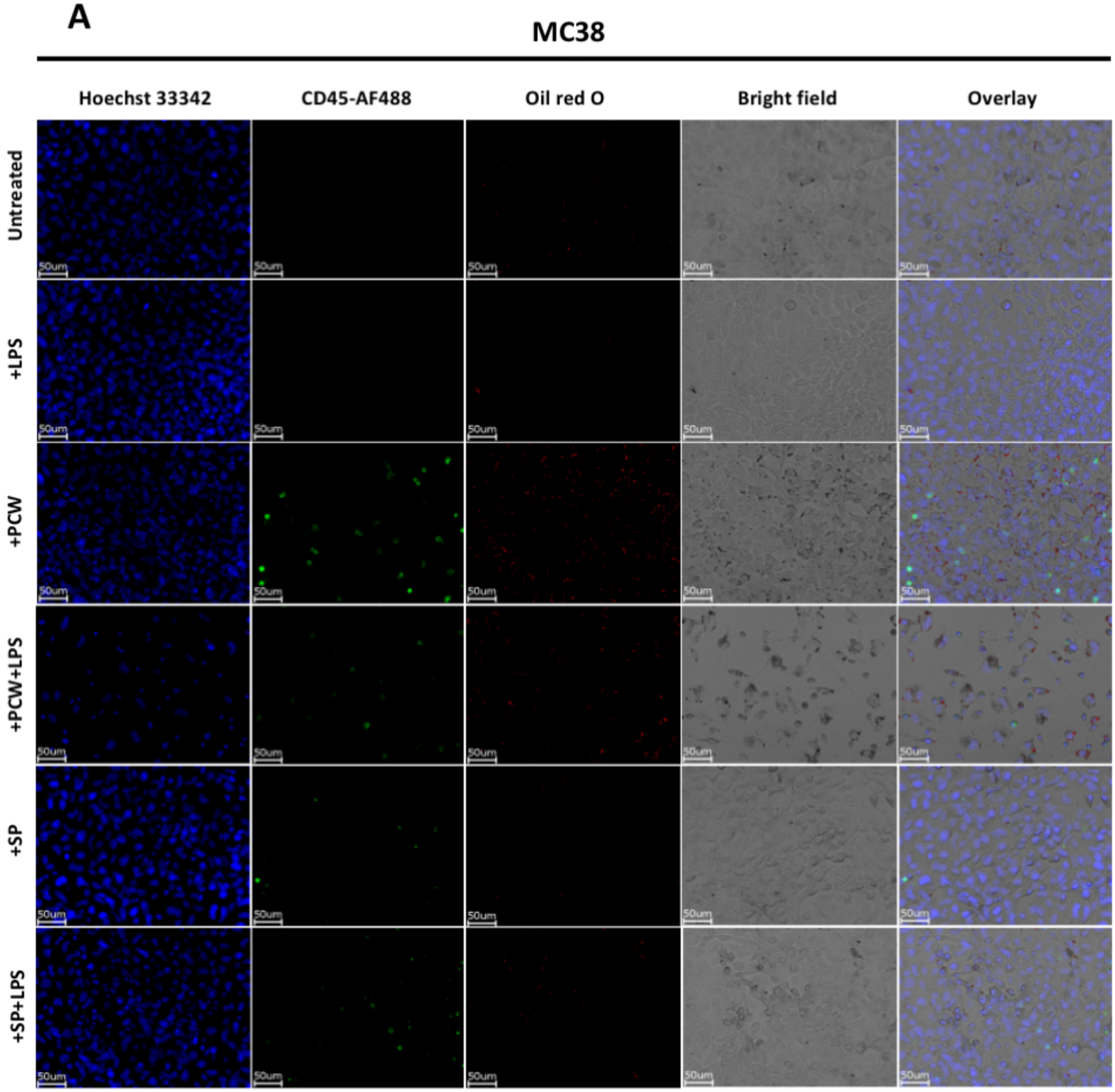
MC38
Hoechst 33342
CD45-AF488
Oil red O
Bright field
Overlay
Untreated
+LPS
+PCW
+PCW+LPS
+SP
+SP+LPS
50um

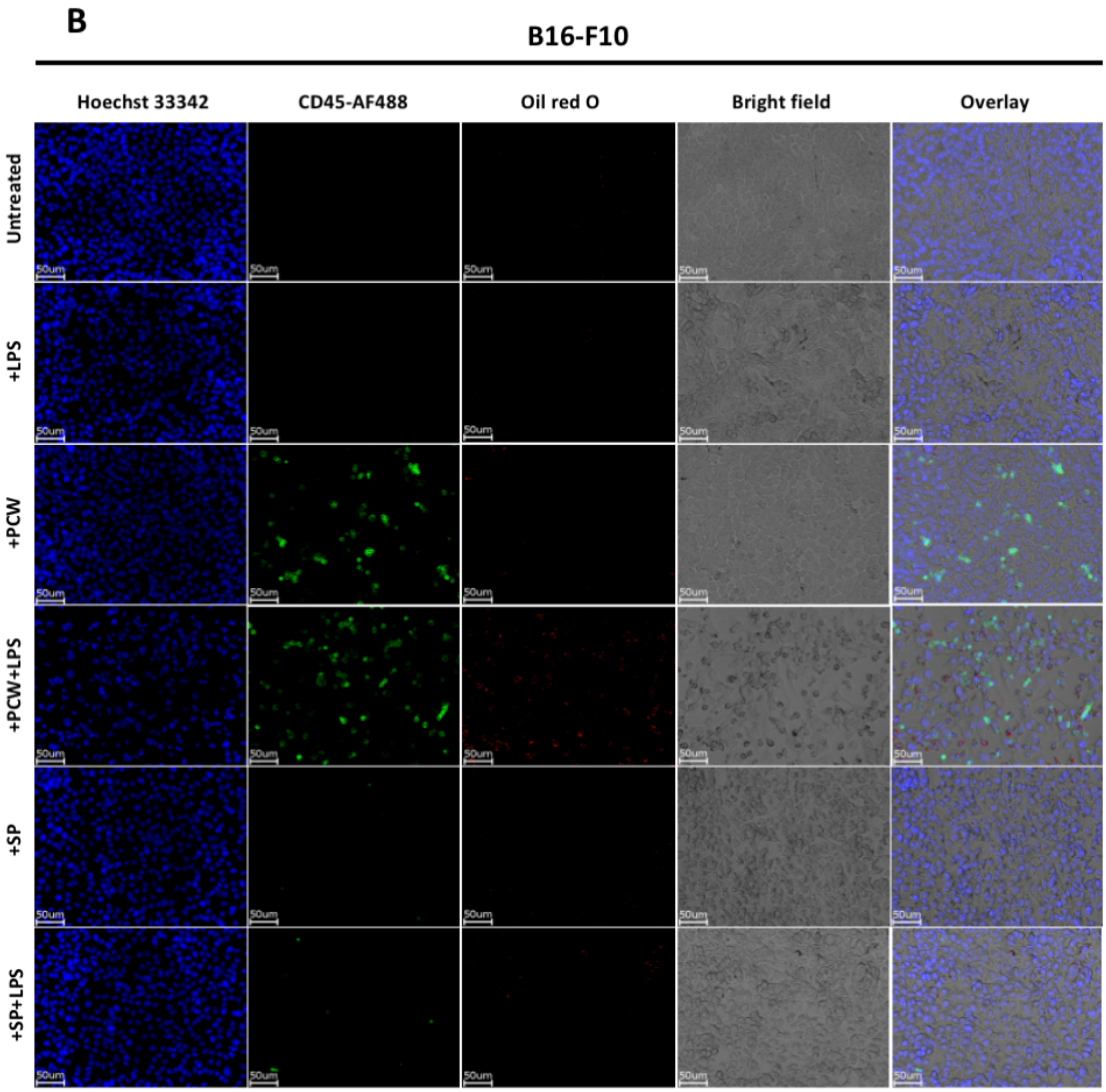

Figure S2. Individual fluorescence channels for representative images of tumor cells cultured under various conditions. (**A**) Representative images of individual channels of MC38 cells cultured alone or co-cultured with PCW cells or splenocytes in the absence or presence of LPS for 72 hours. Cells were stained with Hoechst 33342 for nuclear stain, CD45-AF488 as a leukocyte marker and Oil Red O (ORO) as a lipid stain. Scale bar (white) = 50 µm. Representative images from triplicate wells were taken at 400× magnification. (**B**) Representative images of individual channels of B16F10 cells cultured alone or co-cultured with PCW cells or splenocytes in the absence or presence of LPS for 72 hours. Cells were stained with Hoechst 33342 for nuclear stain, CD45-AF488 as a leukocyte marker and ORO as a lipid stain. Scale bar (white) = 50 µm. Representative images from triplicate wells were taken at 400× magnification.

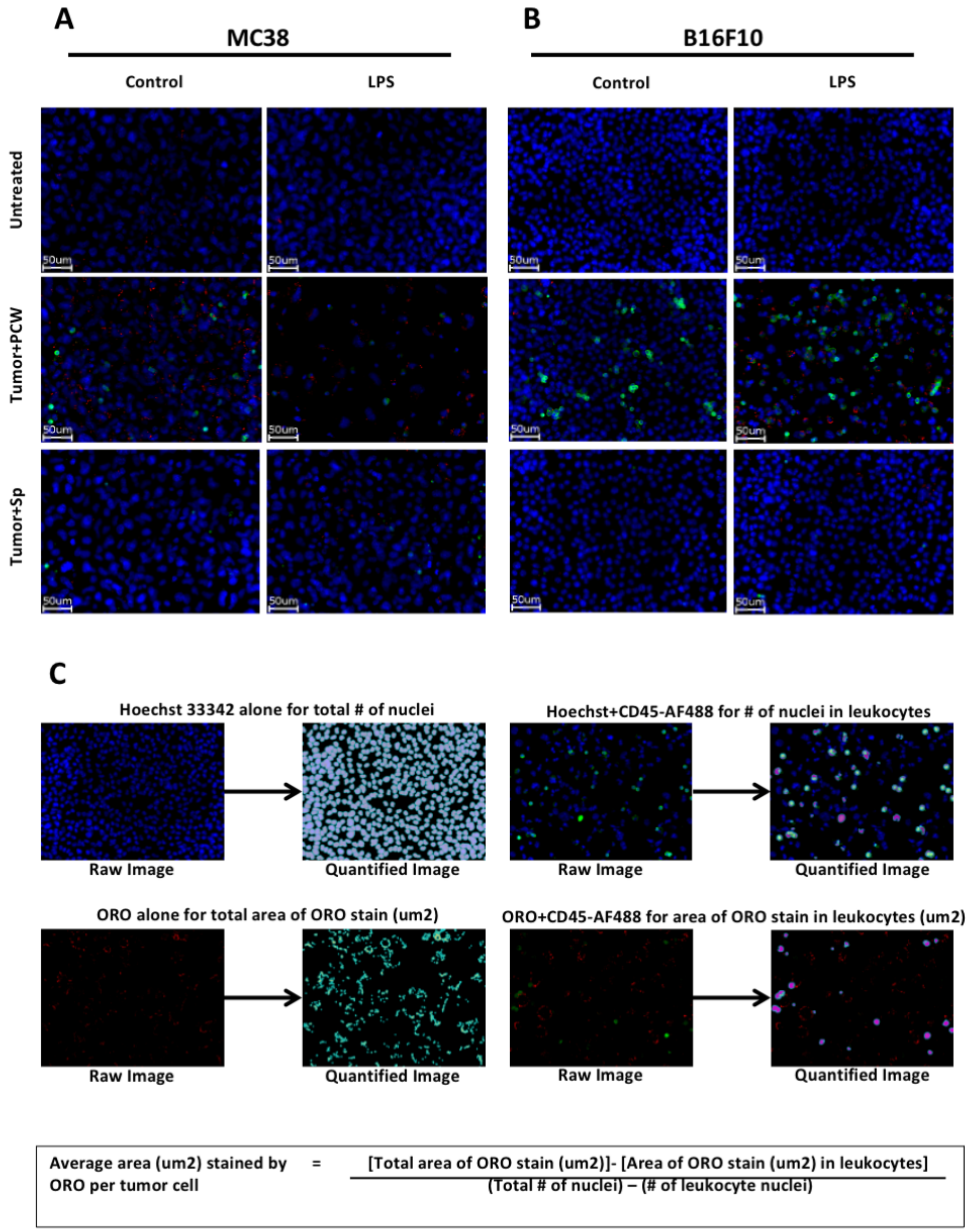

Figure S3. LPS enhances lipoptosis of both MC38 and B16F10 cells. (**A**) MC38 colon cancer cells and (**B**) B16F10 melanoma cells were cultured alone, or co-cultured with PCW cells or splenocytes for 72 hours in the presence and absence of LPS. At 72 hours cells were stained with ORO for lipid stain, Hoechst 33342 as nuclear stain and CD45-AF488 as leukocyte marker. Scale bar (red) = 50 µm. Representative images from triplicate wells were taken at 400× magnification. (**C**) Lipoptosis quantified as the average area of each tumor cell stained by ORO. Areas of Hoechst 33342 staining per field was used to quantify the number of nuclei/field. Areas of Hoechst 33342 staining inside of $CD45^{-}AF488^{+}$ areas was used to quantify the number of leukocytes. Total area of ORO stain in the entire field and inside of $CD45^{-}AF488^{+}$ areas was used to define the amount of lipoptosis in the entire field and in leukocytes, respectively. Average area of ORO stain per tumor cell was used as the final measure of lipoptosis quantification.

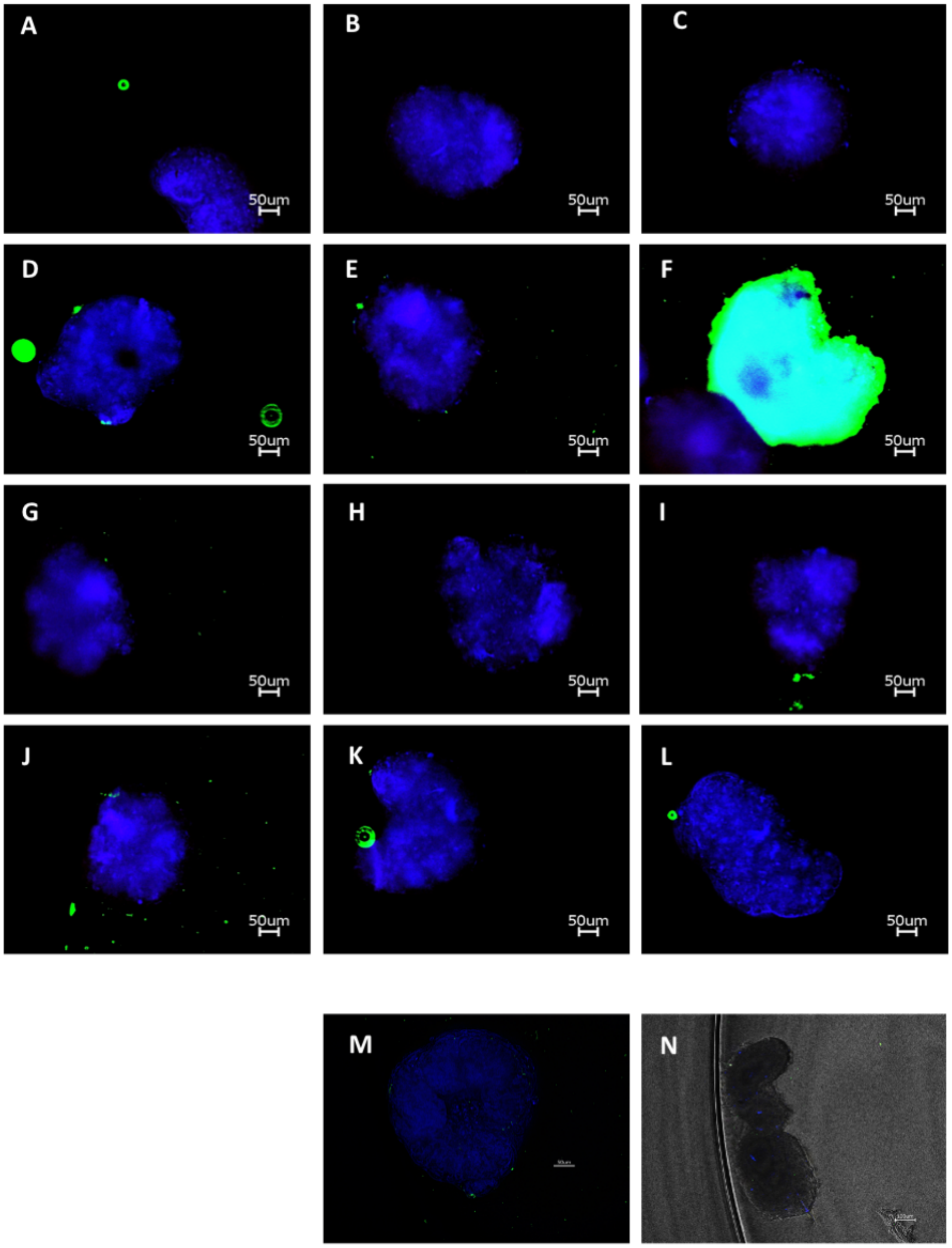

Figure S4. Monoclonal IgM antibodies from L2pB1-derived hybridoma do not recognize MEF spheroids. 3D spheroids prepared using normal mouse embryonic fibroblasts (MEFs) were incubated with the supernatants of each of the L2pB1 hybridoma clones shown in Figure 7 and imaged at 400× magnification (Panel A-L). All hybridoma clones (except the clone shown in Panel F) showed negative staining of MEFs. Scale bars (white) = 50 µm. Results are representative of at least three independent experiments. Panels M and N, imaged at 200× and 100× magnification, respectively, show experimental replicates of 3D MEF spheroids incubated with the hybridoma in Panel F.

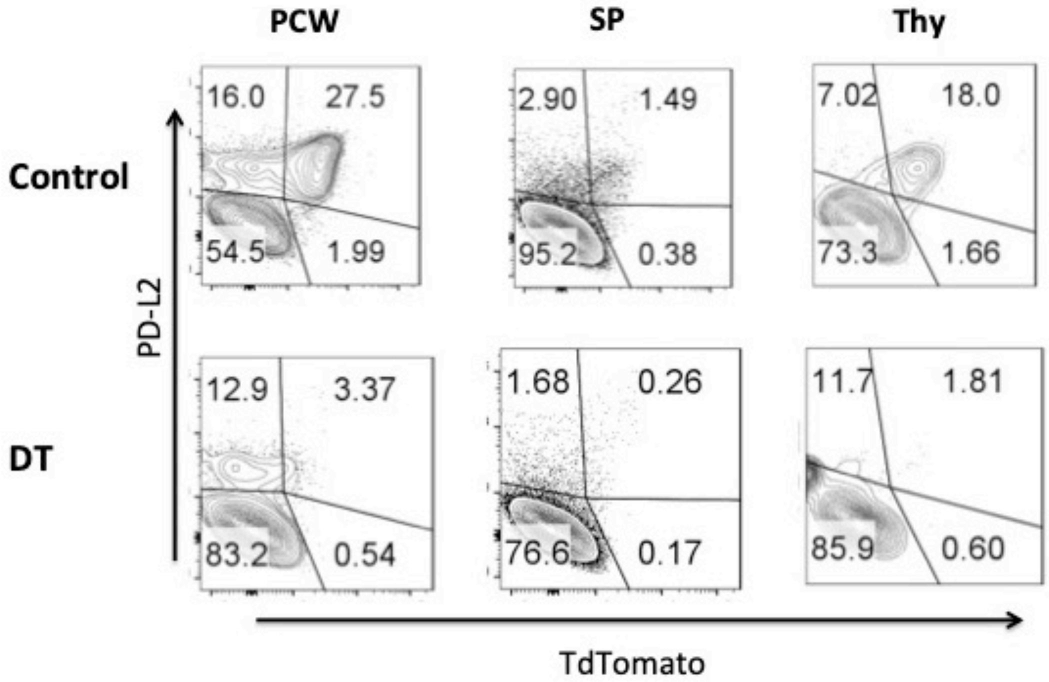

Figure S5. Systemic depletion of L2pB1 cell

CD19-Cre-PZTD mice were i.p. injected with DT for 4 consecutive days. PD-L2 and TdTomato staining in the B-1a cell gate shows the depletion of L2pB1 cells in the peritoneal cavity washout, spleen and thymus. L2pB1 cells are identified here as PD-L2+TdTomato+ double positive cells.

Table S1.

Polyreactivity of L2pB1 hybridoma clones tested with different assays

Clones in figure 7	dsDNA<br><br>reactivity by ELISA	Nucleic acid reactivity by<br><br>ANA assay	Specific protein reactivity by ELISA<br><br>(M7Aa, CHRT1 etc.)	Poly-reactivity by auto-antigen protein microarray<br><br>(Strong, Median, Weak, not tested (NT))
A (32.93.45)	+	Peri-nuclear +	+	NT
B (32.95.81)	+	Peri-nuclear +	+	NT
C Secondary Alone control	NA	NA	NA	NA
D (204.86.44)	+	-	+	Weak
E (14.90.95)	+	Peri-nuclear/cytosol +	+	Weak
F (14.89.35)	+	Peri-nuclear/cytosol +	+	NT
G (14.89.33)	+	Peri-nuclear/cytosol +	+	Weak
H (73.94.45)	+	Peri-nuclear/cytosol +	+	NT
I (79.295.90)	+	Peri-nuclear/cytosol+	+	Median
J (262.84.37)		Nuclear +		Weak
K (287.79.40)	+	-	+	Weak

L (28.95.43)	NT	-	NT	Weak
